# Supplementary material for: Altered Visceral Adipose Tissue Predictors and Women’s Health: A Unicenter Study
Source: Int J Environ Res Public Health. 2022 May 1;19(9):5505. doi: 10.3390/ijerph19095505 (PMC9105488; doi:10.3390/ijerph19095505)
Supplement: Supplementary file 1 [file ijerph-19-05505-s001.zip › ijerph-1668970-supplementary.pdf]

International Journal of
Environmental Research and Public Health

MDPI

Article

Altered Visceral Adipose Tissue Predictors and Women's Health: A Unicenter Study

Supplementary Materials:

Table S1. Reference values used in the present study.

Variable	Reference Value*	Source
Height (m)	Normal: 1.55 – 1.65	[71]
Weight (kg)	Normal: 54.56 – 77.65	[71]
BMI (kg/m^2)	Low weight: < 16.0 - 18.49<br>Normal weight: 18.5 - 24.99<br>Overweight: 25.0 - 29.99 30<br>Obesity: ≥ 30.0	[71]
Subscapular fold (mm)	18 - 24,9 years: 6.5 – 20.0<br>25 - 29,9 years: 6.5 – 23.0<br>30 - 34.9 years: 6.5 – 26.5<br>35 - 39.9 years: 7.0 – 28.5<br>40 - 44.9 years: 6.5 – 28.5<br>45 – 49.0 years: 7.0 – 29.5<br>50 – 54.9 years: 7.0 – 30.0<br>55 – 59.9 years: 7.0 – 31.0	[71]
Triceps fold (mm)	18 - 24,9 years: 9.0 – 24.0<br>25 - 29,9 years: 10.0 – 26.5<br>30 - 34.9 years: 10.5 – 29.5<br>35 - 39.9 years: 11.0 – 30.0<br>40 - 44.9 years: 12.0 – 30.5<br>45 – 49.0 years: 12.0 – 32.0<br>50 – 54.9 years: 12.0 – 32.0<br>55 – 59.9 years: 12.0 – 32.0	[71]
Mid-axillary fold (mm)	Normal: 8.0 – 26.75	[71]
Supra iliac fold (mm)	Normal: 10.03 – 30.0	[71]
Chest fold (mm)	Normal: 8.0 – 22.75	[71]
Abdominal fold (mm)	Normal: 15.65 – 35.0	[71]
Mid-thigh fold (mm)	Normal: 20.0 – 40.0	[71]
Calf fold (mm)	Normal: 9.30 – 28.0	[71]
Arm circumference (cm)	18 - 24,9 years: 22.4 – 29.0<br>25 - 29,9 years: 23.1 – 30.6<br>30 - 34.9 years: 23.8 – 32.0<br>35 - 39.9 years: 24.1 – 32.6<br>40 - 44.9 years: 24.3 – 33.2<br>45 – 49.0 years: 24.2 – 33.5<br>50 – 54.9 years: 24.0 – 33.8<br>55 – 59.9 years: 24.8 – 34.3	[71]
Waist circumference (cm)	Risk: ≥ 80	[71]
Abdominal circumference (cm)	Without risk: < 80<br>High risk: ≥ 80<br>Very high risk: ≥ 88	[71]
Hip circumference (cm)	Normal: 88.65 – 109.0	[71]
Calf circumference (cm)	Normal: 33.0 – 44.0	[71]

Thigh circumference (cm)	Normal: 48.0 – 60.0	[71]
Glucose (mg/dL)	Normal: 70 – 99	Laboratory
Total cholesterol (mg/dL)	Normal: < 190	Laboratory
Triglycerides (mg/dL)	Normal: < 150	Laboratory
HDL (mg/dL)	Normal: > 40	Laboratory
LDL (mg/dL)	Normal: ≤ 129	Laboratory
VLDL (mg/dL)	Normal: > 30	Laboratory
non-HDL (mg/dL)	Normal: ≤ 159	Laboratory
LAP (cm.mmol/L)	Normal: ≤ 26.19	Median[1]
TG/HDL	Normal: ≤ 1.86	Median[1]
TyG	Normal: ≤ 3.67	Median[1]
TyG-BMI	Normal: ≤ 96.60	Median[1]
Waist-to-Hip ratio	Normal: < 0.8	[71]
Jackson Pollock's Fat Percentage (seven-folds) (%)	18 – 29 years: ≤ 31.0<br>30 – 39 years: ≤ 32.0<br>40 – 49 years: ≤ 33.0<br>50 – 59 years: ≤ 34.0	[71]
Muscular mass (kg)	Normal: ≤ 23.16	Median[1]
Visceral adipose tissue (cm²)	Normal: ≤ 105.64	Median[1]
VAT/MM ratio (cm²/kg)	Normal: ≤ 4.54	Median[1]

*Values outside the ranges shown in this table were considered altered. [1] The median of each variable from the data set collected was employed to calculate its measurement's cutoff point.
